# Inpatient Gastroenterology Workups Prior to Transesophageal Echocardiogram: Is It Time for a Change of Heart?

**DOI:** 10.1016/j.gastha.2023.06.001

**Published:** 2023-06-07

**Authors:** RAHUL S. DALAL, DEBORAH C. RUBIN

**Affiliations:** Division of Gastroenterology, Hepatology and Endoscopy, Department of Medicine, Brigham and Women’s Hospital, Harvard Medical School, Boston, Massachusetts; Division of Gastroenterology, Department of Medicine, Washington University School of Medicine, St. Louis, Missouri

In this retrospective cohort study, Mizrahi et al^1^ assessed the utility of inpatient gastroenterology (GI) evaluations prior to transesophageal echocardiogram (TEE). The authors investigated the frequency of GI-related contraindications for TEEs and the utility of GI workup prior to TEE, regardless of the GI team’s interventions. They observed that 57/79 (72%) inpatients underwent an inpatient GI workup prior to TEE, most commonly for dysphagia/odynophagia or a history of GI bleeding or anemia. Workup included an esophagogastroduodenoscopy (EGD) and/or imaging tests. Out of the 49 EGDs performed, all but one resulted in clearance of the patient for TEE.

Patients who had an EGD performed within a year before TEE had 6-fold odds of being cleared by GI without further workup, while patients with active GI symptoms had 0.3-fold odds of being cleared for TEE without further workup. The authors conclude that inpatient workups for TEE procedures lead to wasted utilization of healthcare resources without significant benefits to patients.

The study’s merits include its broad characterization of evaluations for a variety of GI indications, whereas many prior studies have had a more narrow focus, such as cirrhosis and esophageal varices.^2^, ^3^, ^4^ However, the limitations of this work must be appreciated. The small sample of 79 patients is likely not appropriate for multivariable logistic regression due to model overfitting, and conclusions from such analyses cannot be extrapolated to other settings. Additionally, in their regression analysis, the authors found that active GI symptoms at the time of consultation were a significant, independent predictor of further GI workup prior to TEE. Patients with active symptoms may warrant an inpatient GI evaluation regardless of the need for TEE. Therefore, it is not clear that resources were wasted at least for this subset of patients, though there may have been unnecessary delays of TEEs. Furthermore, 79 patients undergoing GI consultations for TEE over an 8-year span represent fewer than 10 patients per year. Therefore, it is unlikely that these consultations inflicted a major resource burden for this health system. This study might benefit from a cost-effectiveness analysis for those undergoing GI workups prior to TEE.

Other studies have more clearly demonstrated waste with regard to GI consultations for TEE clearance. In a recent retrospective analysis of 191 patients, Sack et al determined that cirrhotics with esophageal varices who underwent TEE did not experience overt bleeding, and there was no increased risk of requiring blood transfusions between those with and without endoscopically documented esophageal varices prior to TEE.^2^ By demonstrating that patients with and without known varices carried identical risks for gastrointestinal bleeding post-TEE, which was the primary concern at the time of consultation, justification for GI clearance in this subset of patients is diminished.

As concluded by the authors, many asymptomatic patients likely do not require an inpatient GI evaluation prior to TEE, as they appear to have similar outcomes based on the data presented. However, larger-scale studies are still needed to exclude small differences in potential risks which may have been missed due to type II error in this small cohort. Future research should also investigate the potential cardiac consequences of delaying TEE for an inpatient GI evaluation. This may provide more compelling justification for foregoing a GI workup except for those with new or concerning GI symptoms. Additional research is also needed regarding the burden of unnecessary GI consultations in general and their impact on delivery of more urgent inpatient GI care. This issue is particularly important given the high demand for GI services and the limited resources available to many hospitals.

In conclusion, the study provides some valuable insights into the limited utility of many GI workups prior to TEE, however, the limitations should be considered. Larger studies are needed to address these limitations and provide a more comprehensive evaluation of the impact of GI workups on patient outcomes, healthcare costs, and resource utilization. Until then, the decision to perform an inpatient GI evaluation in a patient undergoing TEE should be made on a case-by-case basis, taking into account the severity of patients’ GI symptoms and the timing and clinical urgency of TEE.

## References

[1] Mizrahi J., Klaychman L., Paradkar A. (2023). Inpatient gastroenterology workup prior to transesophageal echocardiogram is of minimal benefit to patients. Gatro Hep Advances.

[2] Sack J.S., Li M., Zucker S.D. (2021). Bleeding outcomes following transesophageal echocardiography in patients with cirrhosis and esophageal varices. Hepatol Commun.

[3] Pantham G., Waghray N., Einstadter D. (2013). Bleeding risk in patients with esophageal varices undergoing transesophageal echocardiography. Echocardiography.

[4] Spier B.J., Larue S.J., Teelin T.C. (2009). Review of complications in a series of patients with known gastro-esophageal varices undergoing transesophageal echocardiography. J Am Soc Echocardiogr.

